# The changing demography of the cystic fibrosis population: forecasting future numbers of adults in the UK

**DOI:** 10.1038/s41598-020-67353-3

**Published:** 2020-06-30

**Authors:** Ruth H. Keogh, Kamaryn Tanner, Nicholas J. Simmonds, Diana Bilton

**Affiliations:** 1 Department of Medical Statistics, London School of Hygiene and Tropical Medicine, Keppel Street, London, WC1E 7HT UK; 2 National Heart and Lung Institute, Imperial College London, Emmanuel Kaye Building, 1B Manresa Road, London, SW3 6LR UK; 3 Royal Brompton Hospital, Sydney Street, London, SW3 6NP UK

**Keywords:** Cystic fibrosis, Epidemiology, Health services, Statistics, Medical research, Outcomes research

## Abstract

Improvements in management of cystic fibrosis (CF) through specialist centres in the UK have been associated with a step-change in life expectancy. With increasing numbers of adult patients there is a need to review health care provision to ensure it is sufficient to meet future needs. We used UK CF Registry data to project the number of patients aged 16–17 and 18 and older  up to 2030, and numbers therefore requiring specialist adult CF care. Survival modelling was used to estimate age-specific mortality rates. New-diagnosis rates were estimated using diagnoses observed in the Registry and national population figures. Uncertainty in projections was captured through 95% prediction intervals (PI). The number of adults (aged 18 and older) is expected to increase by 28% from 6,225 in 2017 to 7,988 in 2030 (95% PI 7,803–8,169), assuming current mortality rates. If mortality rates improve at the rate seen over recent years, the projected number increases to 8,579 (95% PI 8,386–8,764). The age distribution is also expected to change, with 36% of CF adults being over 40 in 2030, versus 21% in 2017. There is an urgent requirement to review adult CF health care provision, due to both increasing numbers and the changing care needs of an older population.

## Introduction

Improvements in management of cystic fibrosis (CF) delivered in specialist centres in the United Kingdom (UK) have been associated with an overall step-change in CF, from a fatal disease of childhood in the 1960s and 1970s to a disease requiring long-term management through adulthood in the twenty-first century^1^. The UK CF Registry is a key resource for monitoring of patient numbers and outcomes, and according to current registry-based statistics, half of babies born today with CF in the UK are expected to survive beyond the age of 47^2–4^. As a consequence of improving survival, the number of adult CF patients has increased^5,6^. With increasing numbers of adult patients there is a need to review health care provision to ensure it is sufficient to meet the needs of the evolving CF population over the coming years. The UK has provided world leadership in the development of adult specialist centre care, separate from paediatrics^7^ and the European Respiratory Society published a task force report jointly with the European CF Society endorsing the UK approach to specialist care^8^. However, there is growing recognition that care needs may soon need to evolve further, as co-morbidities change and novel effective therapies are introduced^9^.

This work used data from the UK CF Registry and the Office of National Statistics (ONS) to obtain projections of future numbers of adult CF patients (aged 18 and older) up to 2030. We also provide estimates of the number of patients requiring care through specialist adult CF centres, which includes some patients aged 16 and 17. Future numbers depend on the current numbers of adults with CF, numbers of children with CF who will transition to the over 18 age group in time, numbers of new diagnoses, and age-specific mortality rates. The focus is on estimating the number of individuals aged 16 years and older and we present projections of the numbers of patients aged 16–17 and aged 18 and older. To estimate the number who will require care in a specialist adult CF centre we assume that all individuals aged 19 years and older and one third of those aged 16, 17 and 18 are treated in such a centre, as the transition to adult care occurs over this 3 year period. As the transition process is affected by a number of clinical, practical and logistical issues, we took a pragmatic approach by evenly distributing the numbers over this 3 year age range. Alongside the estimates of future patient numbers, we provide 95% prediction intervals to quantify the uncertainty in the estimates. Our work is closely related to that of Burgel et al.^6^, who obtained forecasts of patient numbers in 34 countries in Europe using data from the European CF Society Patient Registry. Their model forecasted an increase in the UK adult CF population (aged 18 and older) from 4,950 in 2010 to 8,876 in 2025, an increase of 76%. We hypothesised that their UK forecasts could be overestimates due to using over-estimates of the rate of new diagnoses, whereby new individuals appearing in the registry were counted as new diagnoses. The study made use of UK data captured over a period in which the UK CF Registry data acquisition methods changed, as a result of changes in the NHS commissioning of care and requirements for data collection, meaning that new individuals captured in the Registry were not necessarily new diagnoses. We avoid this by making use of diagnosis dates as well as dates of birth and death, together with use of UK population forecasts. We compare our findings with those of Burgel et al.

Our results quantify the expected impact of sustained improvements in survival in CF on the population of individuals living with this condition up to 2030, and on the age distribution of the population. The results inform health care provision in the UK, to facilitate planning of service requirements in the presence of a larger CF population and increasing numbers of individuals reaching older ages. Furthermore, the methodology presented provides an approach to obtaining population projections using data from other disease registries and electronic health records, including how to incorporate estimates of uncertainty into the projections.

## Materials and methods

### Data

The primary resource for this study was the UK CF Registry. The UK CF Registry is a national, secure database sponsored and managed by the Cystic Fibrosis Trust^10^. It was established in 1995 and records demographic data and longitudinal health data on nearly all people with CF in the UK, to date capturing data on over 12,000 individuals. The Registry includes > 99% of individuals with CF in the UK^10^, making it a reliable resource for establishing current numbers of individuals with CF and for estimating quantities needed to derive population projections. Data are collected in a standardized way at designated (approximately) annual visits on over 250 variables in several domains, and have been recorded using a centralised database since 2007. This study makes use of dates of birth, diagnosis and death for individuals observed between 1996 and 2017. Dates of birth and death are provided in month–year format and the day was set to be the 15th of the month, following standard practice in survival analyses using UK CF Registry data.

To estimate expected numbers of new diagnoses in future years we required estimates of rates of new diagnoses by age and estimates of the size of the UK population up to 2030. To estimate rates of diagnosis we used numbers of new diagnoses from the UK CF Registry combined with ONS data on the size of the UK population by age up to 2017^11^. ONS projections of the population size from 2018 to 2030 were used to project future numbers of diagnoses^12^.

### Statistical methods: overview

A detailed description of the statistical analysis is given in the Supplementary Material. Here we provide an overview of the approach. The basis of the analysis is to estimate the number of individuals of each age *a* (*a* = 16, 17,…,100) at the start of each calendar year from 2018 to 2030. These are then added together to provide an estimate of the total number of individuals aged 16–17 and the total number aged 18 and older at the start of each year.

The estimated number of patients aged *a* + 1 at the start of 2018 is the observed number aged *a* at the start of 2017 who survive for 1 year, plus the observed number of new diagnoses at age *a* in 2017 who survive to the start of 2018. New diagnoses are all assumed to occur at the start of a given year, and so the number of individuals diagnosed at age *a* in 2017 who survive to the start of 2018 is the number who survive 1 year. To estimate the number of individuals aged *a* + 1 at the start of 2019 we make use of the projected number of individuals aged *a* at the start of 2018 and the projected number of newly diagnosed individuals in 2018, alongside the 1-year survival probabilities. This is repeated year by year up to 2030. Individuals aged 3 years in 2017 will be aged 16 in 2030, and so the analysis requires numbers of individuals aged 3 years and older in 2017. Similarly, we need to take account of new diagnoses aged 4 years and older in 2018, aged 5 and older in 2019, and so on, up to age 15 and older in 2029. We do not need to incorporate information on newborn diagnoses or diagnoses in children aged under 3 years, because no children aged under 3 in 2017 (or later) will reach age 16 by 2030, the end of our forecast period.

### Statistical methods: estimating diagnosis and mortality rates

The analysis requires estimates of the probability of survival to age *a* + 1 conditional on survival to age *a* from each age *a* (*a* = 3,...,100). These probabilities were estimated using the UK CF Registry data using a flexible parametric survival model^13^, following the approach used by Keogh et al.^4^. This made use of dates of birth, diagnosis and death. Age of diagnosis and death were derived and the time scale for the survival analysis was age. Individuals who do not have a recorded date of death were censored at the end of 2017.

The analysis also requires estimated numbers of new diagnoses at each relevant age in each calendar year from 2018 to 2030. Age-specific diagnosis rates were estimated using UK CF Registry data on the number of individuals diagnosed at each age during 2013–2017 and the number of individuals in the UK population over the same period using ONS population data^11^. The number of new diagnoses is small at all ages, so we considered new diagnoses in 5-year age groups, with diagnoses at ages 63 and older being combined due to very small numbers. The probability of diagnosis during a 1-year period was estimated for each age group as the number of diagnoses divided by the number in the UK population. The analysis uses the projected number of individuals in the UK population at each age in years from 2018–2030 multiplied by the diagnosis rates to obtain yearly numbers of new diagnoses at each relevant age. It is assumed that age-specific diagnosis rates will remain similar up to 2030.

The analysis outlined above makes the assumption that age-specific mortality rates will remain the same up to 2030. We also derived population projections under conditions whereby recent decreases in mortality rates continue in the future. Specifically, we estimated the linear downward trend in mortality rates over the 10-year period 2008–2017, and applied this to obtain projections under the assumption that mortality rates continue to decrease at the same rate in the future, or half that rate. This is similar to the approach used by Keogh et al. ^4^ and MacKenzie et al.^14^.

As a sensitivity analysis the analysis was repeated separately in males and females, since females have been shown to have worse survival than males^2,4^. Mortality rates were estimated separately by sex. Diagnosis rates were assumed to be the same in males and females and half of new diagnoses were assumed to be in males and half in females.

We obtained 95% prediction intervals (95% PI) for the projected population numbers using an extended bootstrapping approach^15,16^. The true future numbers are expected to lie within the 95% prediction interval with probability 0.95.

## Results

A total of 12,904 individuals were recorded in the UK CF Registry between its starting year of 1996 and 2017, the most recent year of available data. Over the 10 year period from 2008 to 2017 the number of people aged 18 and older increased by 44% from 4,313 to 6,225, while the number of 16–17 year olds decreased from 627 to 483. Table 1 shows the number of new diagnoses during 2013–2017 by age group alongside the number at those ages in the underlying UK population, and the resulting estimated diagnosis rates. In the youngest age group, 3–7 years, the diagnosis rate was estimated to be 1.54 per million. Diagnosis rates are higher in children and decreased with age. There were no observed new diagnoses after age 81 during 2013–2017 and the new diagnosis rate was assumed to be zero beyond this age.

**Table 1:** Observed numbers of new diagnoses by age group (defined by 5-year age groups, with ages 63 onwards being combined into a single group) from 2013–2017, number of individuals in the UK population during the same period based on ONS statistics, and the ratio (estimated diagnosis rate).

Age group	Number of new diagnoses	Number in the UK population	Estimated diagnosis rate
3–7	31	20,183,682	1.54E−06
8–12	35	18,525,594	1.89E−06
13–17	29	18,289,582	1.59E−06
18–22	24	20,426,605	1.17E−06
23–27	27	22,206,310	1.22E−06
28–32	20	22,020,185	9.08E−07
33–37	37	21,244,044	1.74E−06
38–42	28	20,504,753	1.37E−06
43–47	20	22,723,270	8.80E−07
48–52	12	23,245,834	5.16E−07
53–57	11	21,024,065	5.23E−07
58–62	13	18,257,724	7.12E−07
63–81	19	52,878,448	3.59E−07

**Figure 1:**
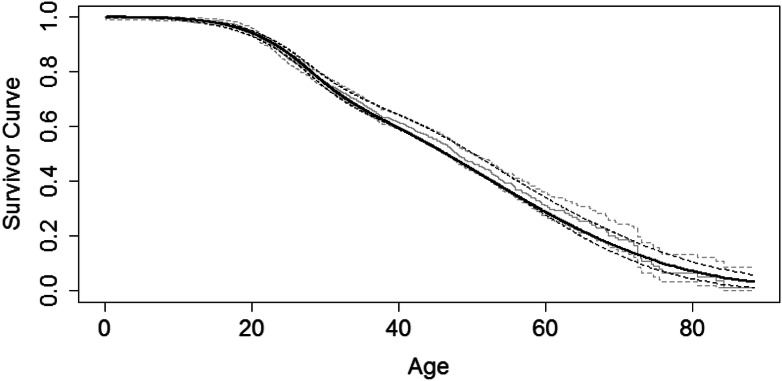
Estimated survivor curve using UK CF Registry data from 2013–2017. The black curve is from the flexible parametric survival model. The grey line shows the Kaplan–Meier estimates for comparison. Dotted lines indicate 95% confidence intervals.

Figure 1 shows the estimated survivor curve obtained using data from 2013–2017. The full specification of the model and corresponding parameter estimates are provided in the Supplementary Materials. This model aligns closely with that of Keogh et al.^4^, but was fitted for the more recent period 2013–2017. Supplementary Table 2 shows the model-based estimated 1-year survival probabilities from ages 3–100, which are used in the projection algorithm.

Table 2 shows the projected numbers of patients aged 16–17 and aged 18 and older for 2018–2030, and corresponding 95% prediction intervals, alongside the observed numbers from 2013–2017. The total number of individuals aged 16–17 is expected to increase by 20% from 483 in 2017 to 578 (95% PI 562–595) in 2030, and the number of individuals aged 18 and older is expected to increase by 28% from 6,225 to 7,988 (95% PI 7,803–8,169) over the same period. The 95% prediction intervals indicate that the actual number of adult CF patients in 2030 is expected lie in the range from 7,803 to 8,169 with probability 0.95. Under the assumption that all individuals aged 19 and older and one-third of patients aged 16–18 require care in an adult CF centre, we estimate that the total number of individuals requiring adult care will increase from 6,212 in 2017 to 7,981 (95% PI 7,797–8,162) in 2030, an increase of 28% (95% PI 26–31%).

**Table 2:** Observed (2013–2017) and projected (2018–2030) numbers of people with CF aged 16–17, aged 18 and older and the expected total number of individuals requiring adult care (calculated as the number aged 19 and older plus one third of the number aged 16–18).

Year	Total aged 16–17		Total aged 18 and older		Total requiring care in an adult centre^a^	
	Number	95% prediction interval	Number	95% prediction interval	Number	95% prediction interval
2013	582	–	5,492	–	5,493	–
2014	559	–	5,700	–	5,688	–
2015	539	–	5,909	–	5,901	–
2016	526	–	6,098	–	6,092	–
2017	483	–	6,225	–	6,212	–
2018	492	(488, 497)	6,390	(6,361, 6,418)	6,379	(6,350, 6,407)
2019	476	(470, 483)	6,505	(6,460, 6,548)	6,518	(6,474, 6,561)
2020	442	(435, 450)	6,669	(6,610, 6,726)	6,635	(6,577, 6,692)
2021	492	(483, 501)	6,759	(6,688, 6,829)	6,788	(6,717, 6,858)
2022	491	(481, 502)	6,881	(6,797, 6,963)	6,886	(6,802, 6,968)
2023	493	(482, 505)	7,011	(6,914, 7,106)	7,008	(6,912, 7,103)
2024	544	(532, 556)	7,124	(7,014, 7,231)	7,147	(7,037, 7,254)
2025	558	(545, 572)	7,248	(7,126, 7,368)	7,265	(7,143, 7,385)
2026	539	(525, 554)	7,402	(7,268, 7,534)	7,390	(7,256, 7,522)
2027	601	(587, 617)	7,532	(7,385, 7,676)	7,554	(7,407, 7,698)
2028	631	(615, 647)	7,659	(7,499, 7,815)	7,690	(7,530, 7,846)
2029	611	(595, 628)	7,841	(7,669, 8,010)	7,825	(7,653, 7,993)
2030	578	(562, 595)	7,988	(7,803, 8,169)	7,981	(7,797, 8,162)

All projected numbers were rounded to the nearest integer, with corresponding 95% prediction intervals

^a^Total number requiring care in an adult centre is the number aged 19 and older plus one third of the number aged 16, 17 or 18.

**Figure 2:**
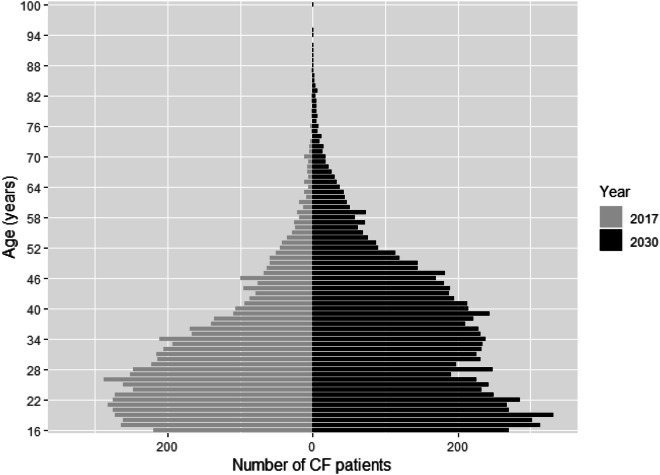
Distribution of ages of people with CF aged 16 and older: based on observed numbers for 2017 and projected numbers for 2030.

Figure 2 compares the observed age-distribution of patients aged 16 and older in 2017 with the projected age distribution in 2030. This indicates a significant change in the shape of the distribution. In 2030 it is expected that a greater percentage of the adult CF population will be older adults. In 2017, 49% of adult (aged 18 and older) were aged over 30, and 21% were aged over 40. In 2030, it is projected that 63% of adults (aged 18 and older) will be aged over 30, and 36% will be aged over 40.

The above results are based on an assumption that current mortality rates will hold in the future. Using data from 2008–2017 it was estimated that age-specific mortality rates decreased by 3% per calendar year [hazard ratio 0.967 (95% CI 0.949, 0.987)]. Table 3 shows the impact on projected numbers if the mortality rates continued to decrease by the same amount each year up to 2030, and if they decreased by half that amount. If mortality rates continue to decrease at the same rate, the projected number of adults in 2030 would be expected to increase by 38% (95% PI 35–41%) to 8,579 (95% PI 8,386–8,764). If mortality rates decreased at half that rate, the projected number projected number of adults in 2030 would be expected to increase by 35% (95% PI 32–38%) to 8,390 (95% PI 8,187–8,588). The projected numbers requiring care in an adult specialist centre would be expected to increase by similar percentages (see Supplementary Table 3).

**Table 3:** Observed (2013–2017) and projected (2018–2030) total numbers of adults (aged 18 and older) with CF, with corresponding 95% prediction intervals for projected numbers: (a) Assuming mortality rates do not change over time; (b) Assuming mortality rates improve at half the rate as during 2008–2017; (c) Assuming mortality rates improve at the same rate as during 2008–2017.

	(a) Assuming mortality rates do not change		(b) Assuming mortality rates improve at half the rate as during 2008–2017		(c) Assuming mortality rates improve at the same rate as during 2008–2017	
Year	Number	95% prediction interval	Number	95% prediction interval	Number	95% prediction interval
2013	5,492	–	5,492	–	5,492	–
2014	5,700	–	5,700	–	5,700	–
2015	5,909	–	5,909	–	5,909	–
2016	6,098	–	6,098	–	6,098	–
2017	6,225	–	6,225	–	6,225	–
2018	6,390	(6,361, 6,418)	6,405	(6,376, 6,433)	6,407	(6,378, 6,435)
2019	6,505	(6,460, 6,548)	6,538	(6,492, 6,582)	6,544	(6,498, 6,588)
2020	6,669	(6,610, 6,726)	6,723	(6,661, 6,781)	6,735	(6,674, 6,793)
2021	6,759	(6,688, 6,829)	6,835	(6,759, 6,908)	6,856	(6,781, 6,927)
2022	6,881	(6,797, 6,963)	6,982	(6,893, 7,069)	7,013	(6,924, 7,098)
2023	7,011	(6,914, 7,106)	7,141	(7,036, 7,241)	7,184	(7,081, 7,281)
2024	7,124	(7,014, 7,231)	7,284	(7,166, 7,399)	7,342	(7,226, 7,452)
2025	7,248	(7,126, 7,368)	7,442	(7,309, 7,571)	7,517	(7,387, 7,639)
2026	7,402	(7,268, 7,534)	7,633	(7,485, 7,774)	7,726	(7,584, 7,861)
2027	7,532	(7,385, 7,676)	7,801	(7,640, 7,957)	7,915	(7,760, 8,062)
2028	7,659	(7,499, 7,815)	7,969	(7,794, 8,139)	8,106	(7,938, 8,266)
2029	7,841	(7,669, 8,010)	8,196	(8,007, 8,380)	8,358	(8,178, 8,531)
2030	7,988	(7,803, 8,169)	8,390	(8,187, 8,588)	8,579	(8,386, 8,764)

Breaking the results down by sex (Supplementary Table 4), the number of adult females with CF is expected to increase by 29% from 2,798 in 2017 to 3,619 (95% PI 3,497–3,739) in 2030, and the number of adult males is estimated to increase by 27% from 3,427 to 4,368 (95% PI 4,246–4,481) over the same time period. The number of females requiring specialist adult care is expected to increase by 35% from 2,792 in 2017 to 3,761 in 2030 (95% PI 3,667–3,856), and the number of males is by 23% from 3,419 to 4,220 (95% PI 4,114–4,324).

## Discussion

We have used UK CF Registry data to obtain projections of numbers of CF patients aged 16 years and older up to 2030. Results show that the expected number of adults (aged 18 and older) with cystic fibrosis is expected to increase by 28% from 6,212 in 2017 to 7,981 in 2030 (95% PI 7,797–8,162). This assumes no improvements in mortality rates. If mortality rates continue to improve at the rate seen over the recent 10 year period, the number would increase to 8,572 (95% PI 8,379–8,756), which is a 38% increase. We also showed how the age distribution of people with CF is expected to change in the coming years, such that there is expected to be a greater proportion of patients at older ages.

As noted in the introduction, our work is closely related to that of Burgel et al.^6^, who obtained forecasts of patient numbers in 34 countries in Europe using data from the European CF Society Patient Registry. As we had hypothesised, our estimates of future adult patient numbers are lower than those obtained by Burgel et al., and this is thought to be due to their counting of new individuals appearing in the registry during a period of improving data capture as new diagnoses. They forecasted 8,876 adult CF patients in the UK in 2025, compared with our estimate of 7,248 (95% PI 7,126, 7,368). The key difference between the two studies is that we made use of diagnosis dates observed in the Registry and UK population forecasts to estimate diagnosis rates, whereas Burgel et al. did not have access to this information and assumed that new individuals observed in the data in a given year were due to new diagnoses. Our methodology differed slightly: they estimated age-specific mortality rates non-parametrically, compared with our flexible parametric approach. This is not thought to have had a major impact on results as the methodological approaches are conceptually similar, involving estimation of how the population flows from one year to the next by estimating how many individuals flow into and out of the population each year. Burgel et al.^17^ updated their earlier results for France, including alterations to account for possible over-estimates of new diagnoses in the earlier work, which was attributed in part to changes in the population coverage of the registry data.

A major strength of our analysis is that we had access to dates of birth, diagnosis and death from the UK CF Registry from 1996 up to the end of 2017. Strengths of our analytical approach are that we made use of ONS statistics to obtain realistic diagnosis rate estimates and that we obtained prediction intervals to quantify the uncertainty in our estimates. Furthermore, we performed sensitivity analyses to assess the potential impact of future improvements in mortality rates. Limitations are that UK population forecasts are made up of different ethnic groups who are at different risk of CF due to genetic differences, and hence the denominator used to estimate diagnosis rates may have been too high. We also assumed that age-specific diagnosis rates will remain similar up to 2030. Diagnosis rates at older ages could, however, increase gradually over time with better diagnostic techniques and more extensive genotyping identifying more individuals with atypical CF. Our assumptions around diagnosis rates are not thought to have had an important impact on the results, since the survival of existing people already known to have CF is the main driver behind the increased projected numbers, rather than new diagnoses.

We did not account in our analysis for the impact of new and future CFTR modulators on survival. Ivacaftor is now used by at least 7% of the UK CF population since its introduction in 2012^18^. Although improvements in mortality rates for this subset of the population will have been incorporated into our survival model, we did not look separately by genotype in order to obtain the projections. Recently (2019) reimbursement for the modulators lumacaftor/ivacaftor (Orkambi) and tezacaftor/ivacaftor (Symkevi) has been agreed in the UK, providing access to a much greater proportion of patients (> 50%), however the effect these therapies have on survival trends will not have been captured by our analyses. Of greater importance will be the impact of so called ‘triple therapy’ (elexacaftor/ivacaftor/tezacaftor)—this highly effective CFTR modulator therapy produced very impressive results in clinical trials and will be potentially prescribed to up to 90% of the CF population^19,20^. In future work it will be important to stratify analyses by genotype and account for the impact of these new treatments. The statistical methods described in this paper could be used in conjunction with estimates of the impact of CFTR modulators on mortality rates, when they become available, to estimate numbers of future patients accounting for these developments. However, predicting this impact on adults – at least in the medium term—is likely to be very challenging as the majority have established and sometimes advanced, multimorbid, disease, which modulators will likely slow at best, so respiratory failure and the associated high care needs (e.g. non-invasive ventilation) will still develop^21^. An ageing CF population is already bringing new challenges, such as increasing diabetes, liver disease and cancer^22–24^. We do not yet know the long-term effect CFTR modulators will have on these. Therefore, the need for specialist care is going to continue to be very relevant but will clearly need to evolve in these changing times.

Data from the 2017 UK CF Registry Annual Report^2^ show that care was provided for adults in 26 specialist centres. Our findings suggest an urgent requirement to review health care provision for adults with CF in the UK as numbers increase, disease characteristics change and new therapies are introduced. It will be important to ensure there is capacity to transition more patients from paediatric care, but perhaps more importantly will be the ability to care for a growing proportion of older, increasingly complicated adults, and also perhaps a more stable younger cohort who will predominantly require outpatient-based care. Innovative technologies with remote monitoring may also be important here. With increasing numbers of patients, many of whom will lead busy active lives despite their comorbidities, we believe these data provide the vital impetus to act now to ensure care provision has the capacity and is fit for purpose for the changing needs of this adult CF population.

## Supplementary information

Supplementary Information 1.

Supplementary Information 2.

## Data Availability

This work used anonymized data from the UK Cystic Fibrosis Registry, which has Research Ethics Approval (REC ref: 07/Q0104/2). The use of the data was approved by the Registry Research Committee (Data Request Reference 349). Data are available following application to the Registry Research Committee. https://www.cysticfibrosis.org.uk/the-work-we-do/uk-cf-registry/apply-for-data-from-the-uk-cf-registry.
