# Supplementary material for: The changing demography of the cystic fibrosis population: forecasting future numbers of adults in the UK
Source: Sci Rep. 2020 Jun 30;10:10660. doi: 10.1038/s41598-020-67353-3 (PMC7327064; doi:10.1038/s41598-020-67353-3)
Supplement: Supplementary file 1 — Supplementary Information 1. [file 41598_2020_67353_MOESM1_ESM.pdf]

The changing demography of the cystic fibrosis population: Forecasting future numbers of adults in the UK

Supplementary Materials: Statistical Methods

Ruth H. Keogh, Kamaryn Tanner, Nicholas J. Simmonds, Diana Bilton

1 Statistical methods: Flexible parametric survival model

Flexible parametric survival models were proposed by Royston and Parmar (2002). We use a flexible parametric survival model in which the transformed probability of survival beyond age a, $\log(-\log \Pr(T > a))$, is modelled using a restricted cubic spline with m internal knots $k_1, \ldots, k_m$ and boundary knots k_{min} and k_{min},

$$\log(-\log \Pr(T > a)) = \gamma_0 + \gamma_1 \log a + \gamma_2 v_1(\log a) + \ldots + \gamma_{m+1} v_m(\log a) \quad (1)$$

where

$$v_j(\log a) = (\log a - k_j)_+^3 - \frac{k_{max} - k_j}{k_{max} - k_{min}} (\log a - k_{min})_+^3 - \frac{k_j - k_{min}}{k_{max} - k_{min}} (\log a - k_{max})_+^3$$

and where $(\log a - k_j)_+ = \max(0, \log a - k_j)$.

For the extended analysis which estimates a linear trend in mortality rates with calendar time, the form of the flexible model is

$$\log(-\log \Pr(T > a + 1 | T > a, y(a))) = \gamma_0 + \gamma_1 \log a + \gamma_2 v_1(\log a) + \ldots + \gamma_{m+1} v_m(\log a) + \eta y(a) \quad (2)$$

where $y(a)$ denotes the calendar year at age a.

The first model was also fitted with a term for sex (male (1) versus female (0)):

$$\log(-\log \Pr(T > a | sex)) = \gamma_0 + \gamma_1 \log a + \gamma_2 v_1(\log a) + \ldots + \gamma_{m+1} v_m(\log a) + \beta \text{sex} \quad (3)$$

The models (1) and (3), without calendar year, was fitted using data from 2013-2017. Model (2), including the calendar year term, was fitted using data from 2008-2017. Age at death was calculated based on dates of birth and death. For individuals who did not die the age of censoring was calculated as the age at 31st December 2017. There was left truncation at the later of the age of diagnosis and the age at the start of 2013 (models (1) and (3)) or the age at the start of 2008 (model (2)). All analyses were performed using R. Flexible parametric survival models were fitted using the flexsurv package (Jackson 2016). For model (1) and (3) we used $m = 3$ and the knot positions derived using the default settings in flexsurv were $-1.789708, 3.174511, 3.414974, 3.728103, 4.434785$. The estimated survivor curve is shown in Figure 1 in the main text. Supplementary Figure 1 shows the curves for males and females from model (3). Model (2), with the calendar year term, was fitted using data from 2008-2017 we used $m = 4$ and the knot positions derived using the default settings in flexsurv were $-1.789708, 3.071933, 3.276532, 3.465736, 3.745597, 4.434785$. Supplementary Table 1 shows the parameter estimates and corresponding 95% confidence intervals from the flexible parametric models.

Supplementary Table 1: Results from flexible parametric survival models.

	Model (1)		Model (2)		Model (3)	
Parameter	Estimate	95% CI	Estimate	95% CI	Estimate	95% CI
γ_0	-9.204	(-10.563, -7.846)	-9.644	(-10.741, -8.547)	-9.073	(-10.425, -7.721)
γ_1	0.387	(-0.360, 1.134)	0.303	(-0.286, 0.892)	0.383	(-0.357, 1.124)
γ_2	-8.205	(-10.004, -6.406)	-10.224	(-13.085, -7.364)	-8.191	(-9.998, -6.384)
γ_3	15.458	11.846, 19.071)	17.512	(9.880, 25.145)	15.393	(11.764, 19.023)
γ_4	-8.095	(-10.282, -5.908)	-4.834	(-11.712, 2.045)	-8.029	(-10.226, -5.831)
γ_5	-	-	-2.923	(-5.435, -0.412)	-	-
η	-	-	-0.033	(-0.052, -0.013)	-	-
Sex (M vs F)	-	-	-	-	-0.317	(-0.464, -0.169)

2 Statistical methods: obtaining projections

2.1 Notation

Let $n_a^{(2017)}$ denote the number of people with CF aged a in 2017. The aim is to estimate the number of patients aged a ($a = 0, \ldots,$) in year y, where $y = 2018, \ldots, 2030$. We use the following notation:

$n_a^{(y)}$ = No. CF individuals aged a at the start of year y ($y = 2018, \ldots, 2030$)
$N_a^{(y)}$ = No. individuals aged a at the start of year y in the UK population
$m_a^{(y)}$ = No. CF individuals aged a at the start of year y who survive to the start of year $y+1$ (ie. to age $a+1$)
$q_a^{(y)}$ = No. CF individuals newly diagnosed at age a in year y who survive to the start of year $y+1$ (ie. to age $a+1$)
$\theta_a^{(y)}$ = Probability of survival to age $a+1$ conditional on survival to age a in year y for a person with CF
ϕ_a = Probability of CF diagnosis at age a, among individuals aged a in year y in the UK population

The conditional survival probabilities $\theta_a^{(y)}$, $\Pr(T > a+1 | T > a, y(a))$ are estimated from the flexible survival models described above. However, they could originate from any suitable model, or from a non-parametric analysis. In the standard analysis the $\theta_a^{(y)}$ do not depend on year and are obtained from model (1). When we assume that mortality rates continue to improve at the same rate at which they have during 2008-2017, we obtain the mortality rates at each age in 2017 using model (2) to give $\theta_a^{(2017)}$. These are then multiplied by $\exp(\hat{\eta}) =$ for each year after 2017. The estimated conditional survival probabilities are denoted $\hat{\theta}_a^{(y)}$.

The diagnosis rates ϕ_a are estimated based on number of observed diagnoses in the registry and numbers of individuals in the UK population at each age based on figures from the Office of National Statistics (ONS), using $\hat{\phi}_a = d_{g(a)}^{(2013-2017)} / N_{g(a)}^{(2013-2017)}$, where $d_{g(a)}^{(2013-2017)}$ denotes the number of diagnoses in the age group $g(a)$ of which a is a member during 2013-2017, and $N_{g(a)}^{(2013-2017)}$ is the total number of individuals in the UK population in the age group $g(a)$ of which a is a member during 2013-2017. Diagnosis rates are assumed to be the same in every year. The UK population numbers $N_a^{(y)}$ needed for future years will be obtained from published ONS figures. The ONS do not provide variance estimates and so these numbers will be assumed known.

$m_a^{(y)}$ and $q_a^{(y)}$ are binomial random variables:

$$m_a^{(y)} | n_{a-1}^{(y-1)} \sim \text{Bin}\left(n_{a-1}^{(y-1)}, \theta_{a-1}^{(y-1)}\right)$$

and

$$q_a^{(y)} | N_{a-1}^{(y-1)} \sim \text{Bin}\left(N_{a-1}^{(y-1)}, \phi_{a-1}\theta_{a-1}^{(y-1)}\right).$$

2.2 Projected numbers

The number of CF individuals aged a at the start of year 2018 is the number who were aged $a-1$ in 2017 and who survive to 2018 *plus* the number of individuals newly diagnosed at age $a-1$ in 2017 who survive to 2018. Using the above notation, this can be written as

$$n_a^{(2018)} = m_{a-1}^{(2017)} + q_{a-1}^{(2017)} \tag{4}$$

It follows that the expectation of $n_a^{(2018)}$ is

$$\begin{aligned} E\left(n_a^{(2018)}\right) &= E\left(m_{a-1}^{(2017)}; \theta_{a-1}^{(y-1)}\right) + E\left(q_{a-1}^{(2017)}\right) \\ &= n_{a-1}^{(2017)}\theta_{a-1}^{(y-1)} + N_{a-1}^{(2017)}\phi_{a-1}\theta_{a-1}^{(y-1)} \end{aligned} \tag{5}$$

A general formula for the number of CF individuals aged a at the start of year y ($y = 2018, \ldots, 2030$) is

$$n_a^{(y)} = m_{a-1}^{(y-1)} + q_{a-1}^{(y-1)} \tag{6}$$

The expectation of $n_a^{(y)}$ is

$$\begin{aligned} E\left(n_a^{(y)}\right) &= E\left(m_{a-1}^{(y-1)} + q_{a-1}^{(y-1)}\right) \\ &= E\left\{E\left(m_{a-1}^{(y-1)} | n_{a-1}^{(y-1)}\right)\right\} + N_{a-1}^{(y-1)}\phi_{a-1}\theta_{a-1}^{(y-1)} \\ &= E\left(n_{a-1}^{(y-1)}\right)\theta_{a-1}^{(y-1)} + N_{a-1}^{(y-1)}\phi_{a-1}\theta_{a-1}^{(y-1)} \end{aligned} \tag{7}$$

The numbers $n_a^{(y)}$ refer to the start of year y. The total number of individuals alive at a given age at any time in year y additionally includes those who are diagnosed in year y. We let $t_a^{(y)} = n_a^{(y)} + d_a^{(y)}$ where $d_a^{(y)}$ is the number diagnosed at age a in year y. The number diagnosed has a binomial distribution $d_a^{(y)} | N_a^{(y)} \sim \text{Bin}\left(N_a^{(y)}, \phi_a\right)$ and so $E\left(t_a^{(y)}\right) = E\left(n_a^{(y)}\right) + N_a^{(y)}\phi_a$.

Our primary results are for numbers combined across ages and we are specifically interested in $\sum_{a=16}^{17} t_a^{(y)}$, $\sum_{a=18}^{100} t_a^{(y)}$ and $\sum_{a=19}^{100} t_a^{(y)} + \frac{1}{3}\sum_{a=16}^{18} t_a^{(y)}$, ($y = 2018, \ldots, 2030$), where the last sum is based on the assumption that all individuals aged 19 and older plus one third of individuals aged 16-18 receive care in a specialist adult centre.

The estimated expected numbers reported in the results for years $y = 2018, \ldots, 2030$ are: (i) the expected number of individuals aged 16-17, $\hat{E}\left(\sum_{a=16}^{17} t_a^{(y)}\right)$, (ii) the expected number of individuals aged 18 and older, $\hat{E}\left(\sum_{a=18}^{100} t_a^{(y)}\right)$, and (iii) the expected total number of individuals requiring care in a specialist adult CF centre, $\hat{E}\left(\sum_{a=19}^{100} t_a^{(y)}\right) + \frac{1}{3}E\left(\sum_{a=16}^{18} t_a^{(y)}\right)$. The hats on the expectations, $\hat{E}(\cdot)$, denote that the parameters $\theta_a^{(y)}$, ϕ_a are replaced by their estimates $\hat{\theta}_a^{(y)}$, $\hat{\phi}_a$

2.3 Prediction intervals for projected numbers

We obtained 95% prediction intervals for the totals $\sum_{a=16}^{17} t_a^{(y)}$, $\sum_{a=18}^{100} t_a^{(y)}$, and $\sum_{a=19}^{100} t_a^{(y)} + \frac{1}{3}\sum_{a=16}^{18} t_a^{(y)}$. Prediction intervals are different from confidence intervals and are wider than confidence intervals. The 95% prediction interval is the range within which the actual number of individuals is expected to lie with 95% probability. Confidence intervals for the expected totals $\hat{E}\left(\sum_{a=16}^{17} t_a^{(y)}\right)$, $\hat{E}\left(\sum_{a=18}^{100} t_a^{(y)}\right)$, and $\hat{E}\left(\sum_{a=19}^{100} t_a^{(y)}\right) + \frac{1}{3}E\left(\sum_{a=16}^{18} t_a^{(y)}\right)$ would take into account the uncertainty in estimation of the parameters $\theta_a^{(y)}$, ϕ_a. A prediction interval refers to the *actual* totals ($\sum_{a=16}^{17} t_a^{(y)}$, $\sum_{a=18}^{100} t_a^{(y)}$, $\sum_{a=19}^{100} t_a^{(y)} + \frac{1}{3}\sum_{a=16}^{18} t_a^{(y)}$) rather than the *expected* totals and hence additionally accounts for the uncertainty in the numbers even for fixed values of parameters $\theta_a^{(y)}$, ϕ_a. That is, even if $\theta_a^{(y)}$, ϕ_a were known exactly rather than being estimated, there would still be uncertainty about the actual totals we will see, and this is what is captured in the prediction interval.

A nested resampling procedure, which is related to bootstrapping, was used to obtain the 95% prediction intervals. We describe this approach to obtain the 95% prediction interval for a total $\sum_a t_{a\in\mathcal{A}}^{(y)}$, where $\mathcal{A}$ denotes some set of ages of interest. This approach is a modified version of that described by Davison and Hinkley (1997, Section 6.3.3), and the first step uses the bootstrapping approach described by Mandel (2013). The steps are as follows:

1. Obtain J draws of the parameters $\theta_a^{(y)}$ and ϕ_a ($a = 3, \ldots, 100; y = 2017, \ldots, 2030$) from their approximated posteriors and denote the draws $\theta_a^{(y),[j]}$, $\phi_a^{[j]}$, $j = 1, \ldots, J$.

   (a) To obtain posterior draws of $\theta_a^{(y)}$ ($a = 3, \ldots, 100; y = 2017, \ldots, 2030$), we first obtain J posterior draws of the set of parameters of the flexible parametric survival model by sampling from a multivariate normal distribution with mean vector given by the parameter estimates and variance-covariance matrix given by the estimated variance covariance matrix for the parameter estimates. The draws $\theta_a^{(y),[j]}$ ($j = 1, \ldots, J$) are obtained directly by obtaining the conditional survival probabilities from the flexible parametric model but with the parameter values set to the jth set of draws.

   (b) Because the diagnosis rates are close to zero it was not considered appropriate to obtain draws from a normal distribution using the central limit theorem. Instead we obtained J draws $d_{g(a)}^{(2013-2017),[j]}$ ($j = 1, \ldots, J$)($a = 3, \ldots, 100$) from a binomial distribution $\text{Bin}\left(N_{g(a)}^{(2013-2017)}, \hat{\phi}_a\right)$ and then defined $\phi_a^{[j]} = d_{g(a)}^{(2013-2017),[j]} / N_{g(a)}^{(2013-2017)}$.

2. For each j, using the parameter draws $\theta_a^{(y),[j]}$ and $\phi_a^{[j]}$ ($a = 3, \ldots, 100; y = 2017, \ldots, 2030$), for $k = 1, \ldots, K$:

   (a) For year $y = 2018$, and sequentially by age ($a = 3, \ldots, 100$), obtain a draw $m_a^{(y),[j,k]}$ from a binomial distribution $\text{Bin}\left(n_{a-1}^{(y-1),[j,k]}, \theta_{a-1}^{(y-1),[j]}\right)$, and a draw $q_a^{(y),[j,k]}$ from a binomial distribution $\text{Bin}\left(N_{a-1}^{(y-1)}, \phi_{a-1}^{[j]}\theta_{a-1}^{(y-1),[j]}\right)$, and a draw $d_a^{(y),[j,k]}$ from a binomial distribution $\text{Bin}\left(N_a^{(y)}, \phi_a^{[j]}\theta_{a-1}^{(y),[j]}\right)$

   (b) Calculate $n_a^{(y),[j,k]} = m_{a-1}^{(y-1),[j,k]} + q_{a-1}^{(y-1),[j,k]}$ and $t_a^{(y),[j,k]} = n_a^{(y),[j,k]} + d_a^{(y),[j,k]}$.

   (c) Repeat steps (a) and (b) sequentially for years $y = 2019, \ldots, 2030$

3. For each year $y = 2018, \ldots, 2030$ calculate $\sum_{a\in\mathcal{A}} t_a^{(y),[j,k]}$ for $j = 1, \ldots, J; k = 1, \ldots, K$.

4. The estimated 95% prediction interval for $\sum_{a\in\mathcal{A}} t_a^{(y)}$ is given by the the 2.5th and 97.5th percentiles of the $J \times K$ values $\sum_{a\in\mathcal{A}} t_a^{(y),[j,k]}$.

We used $J = 1000$ and $K = 100$ for this procedure.

References

Davison AC, Hinkley DV. *Bootstrap methods and their appliation.* Cambridge University Press, 1997.

Jackson C. flexsurv: A Platform for Parametric Survival Modeling in R. *Journal of Statistical Software* 2016; 70 (8): doi: 10.18637/jss.v070.i08.

Mandel M. Simulation-based confidence intervals for functions with complicated derivatives. *The American Statistician* 2013; 67: 7681.

Royston P, Parmar MKB. Flexible parametric proportional-hazards and proportional-odds models for censored survival data, with application to prognostic modelling and estimation of treatment effects. *Statistics in Medicine* 2002; 21: 21752197.
