# Supplementary material for: The changing demography of the cystic fibrosis population: forecasting future numbers of adults in the UK
Source: Sci Rep. 2020 Jun 30;10:10660. doi: 10.1038/s41598-020-67353-3 (PMC7327064; doi:10.1038/s41598-020-67353-3)
Supplement: Supplementary file 2 — Supplementary Information 2. [file 41598_2020_67353_MOESM2_ESM.pdf]

The changing demography of the cystic fibrosis population: Forecasting future numbers of adults in the UK

Supplementary Tables 2-4

Ruth H. Keogh[1*], Kamaryn Tanner[1], Nicholas J. Simmonds[2,3], Diana Bilton[2,3]

1. Department of Medical Statistics, London School of Hygiene & Tropical Medicine, Keppel Street, London, UK, WC1E 7HT.

2. National Heart and Lung Institute, Imperial College London, Emmanuel Kaye Building, 1B Manresa Road, London, UK, SW3 6LR.

3. Royal Brompton Hospital, Sydney Street, London, UK, SW3 6NP.

Please note: Supplementary Table 1 and a statistical appendix can be found in an additional file.

Supplementary Table 2: Estimates of 1-year survival probabilities from each age (0-100) obtained from the flexible parametric survival model (Figure 1). The projection algorithm uses the probabilities from age 3 onwards.

Age	1-year survival probability	Age	1-year survival probability	Age	1-year survival probability	Age	1-year survival probability
0	0.999868						
1	0.999859	26	0.975149	51	0.964023	76	0.915109
2	0.999787	27	0.973743	52	0.962393	77	0.912903
3	0.999693	28	0.973053	53	0.960731	78	0.91069
4	0.999572	29	0.973214	54	0.959038	79	0.908471
5	0.999421	30	0.974289	55	0.957314	80	0.906247
6	0.999233	31	0.975807	56	0.955559	81	0.90402
7	0.999004	32	0.977285	57	0.953774	82	0.901792
8	0.998726	33	0.978613	58	0.951959	83	0.899564
9	0.998394	34	0.979717	59	0.950114	84	0.897336
10	0.997998	35	0.980549	60	0.94824	85	0.895097
11	0.997531	36	0.981078	61	0.946338	86	0.89284
12	0.996983	37	0.981285	62	0.944408	87	0.890563
13	0.996343	38	0.98116	63	0.942452	88	0.888268
14	0.995602	39	0.980699	64	0.94047	89	0.885955
15	0.994746	40	0.979896	65	0.938464	90	0.883623
16	0.993763	41	0.978751	66	0.936434	91	0.881273
17	0.992638	42	0.977397	67	0.934382	92	0.878905
18	0.991355	43	0.976012	68	0.932309	93	0.87652
19	0.9899	44	0.974607	69	0.930215	94	0.874117
20	0.988253	45	0.973177	70	0.928103	95	0.871697
21	0.986396	46	0.971723	71	0.925973	96	0.86926
22	0.984308	47	0.970241	72	0.923827	97	0.866805
23	0.981969	48	0.968731	73	0.921666	98	0.864335
24	0.979453	49	0.967192	74	0.919492	99	0.861847
25	0.977109	50	0.965623	75	0.917306	100	0.859344

Supplementary Table 3. Observed (2013-2017) and projected (2018-2030) numbers of people aged 16-17, aged 18 and older, and the expected total number of individuals requiring adult care (calculated as the number aged 19 and older plus one third of the number aged 16-18) under two scenarios for future mortality rates. All projected numbers were rounded to the nearest integer, with corresponding 95% prediction intervals.

(a) Assuming mortality rates improve in the future at half the rate that they have during 2008-2017

	Total aged 16-17		Total aged 18 and older		Total requiring care in an adult centre*	
Year	Projected number	95% prediction interval	Projected number	95% prediction interval	Projected number	95% prediction interval
2013	582	-	5492	-	5493	-
2014	559	-	5700	-	5688	-
2015	539	-	5909	-	5901	-
2016	526	-	6098	-	6092	-
2017	483	-	6225	-	6212	-
2018	493	(488, 497)	6405	(6376, 6433)	6396	(6367, 6424)
2019	477	(471, 483)	6538	(6492, 6582)	6558	(6512, 6601)
2020	443	(436, 451)	6723	(6661, 6781)	6701	(6640, 6759)
2021	493	(484, 502)	6835	(6759, 6908)	6885	(6809, 6956)
2022	493	(483, 503)	6982	(6893, 7069)	7018	(6929, 7102)
2023	495	(485, 506)	7141	(7036, 7241)	7181	(7078, 7278)
2024	546	(535, 558)	7284	(7166, 7399)	7365	(7249, 7475)
2025	561	(548, 574)	7442	(7309, 7571)	7534	(7404, 7656)
2026	542	(529, 556)	7633	(7485, 7774)	7713	(7571, 7848)
2027	605	(591, 620)	7801	(7640, 7957)	7937	(7782, 8084)
2028	635	(621, 651)	7969	(7794, 8139)	8137	(7969, 8297)
2029	615	(601, 631)	8196	(8007, 8380)	8342	(8162, 8514)
2030	583	(568, 599)	8390	(8187, 8588)	8572	(8379, 8756)

b) Assuming mortality rates improve in the future at the same rate that they have during 2008-2017

	Total aged 16-17		Total aged 18 and older		Total requiring care in an adult centre*	
Year	Projected number	95% prediction interval	Projected number	95% prediction interval	Projected number	95% prediction interval
2013	582	-	5492	-	5493	-
2014	559	-	5700	-	5688	-
2015	539	-	5909	-	5901	-
2016	526	-	6098	-	6092	-
2017	483	-	6225	-	6212	-
2018	493	(488, 497)	6407	(6378, 6435)	6394	(6365, 6422)
2019	477	(471, 483)	6544	(6498, 6588)	6551	(6506, 6595)
2020	443	(436, 451)	6735	(6674, 6793)	6689	(6628, 6747)
2021	493	(485, 502)	6856	(6781, 6927)	6864	(6789, 6937)
2022	493	(484, 503)	7013	(6924, 7098)	6987	(6898, 7074)
2023	496	(486, 507)	7184	(7081, 7281)	7138	(7034, 7238)
2024	547	(536, 559)	7342	(7226, 7452)	7307	(7189, 7421)
2025	562	(549, 575)	7517	(7387, 7639)	7459	(7326, 7587)
2026	543	(530, 558)	7726	(7584, 7861)	7620	(7473, 7762)
2027	607	(593, 622)	7915	(7760, 8062)	7823	(7662, 7978)
2028	637	(623, 653)	8106	(7938, 8266)	8000	(7825, 8169)
2029	617	(603, 633)	8358	(8178, 8531)	8180	(7991, 8363)
2030	585	(570, 601)	8579	(8386, 8764)	8383	(8180, 8581)

* Total number requiring care in an adult centre is the number aged 19 and older plus one third of the number aged 16, 17 or 18.

Supplementary Table 4. Observed (2013-2017) and projected (2018-2030) numbers of people with CF aged 16-17, aged 18 and older and the expected total number of individuals requiring adult care (calculated as the number aged 19 and older plus one third of the number aged 16-18), separately for males and females. All projected numbers were rounded to the nearest integer, with corresponding 95% prediction intervals.

(a) Males

	Total aged 16-17		Total aged 18 and older		Total requiring care in an adult centre*	
Year	Number	95% prediction interval	Number	95% prediction interval	Number	95% prediction interval
2013	282	-	3052	-	3047	-
2014	301	-	3144	-	3155	-
2015	287	-	3258	-	3256	-
2016	273	-	3359	-	3349	-
2017	244	-	3427	-	3419	-
2018	236	(233, 239)	3521	(3501, 3540)	3495	(3475, 3515)
2019	235	(231, 239)	3576	(3546, 3605)	3563	(3533, 3593)
2020	210	(205, 215)	3657	(3618, 3695)	3606	(3568, 3644)
2021	233	(228, 239)	3708	(3660, 3753)	3672	(3626, 3717)
2022	251	(244, 258)	3759	(3703, 3813)	3716	(3662, 3768)
2023	259	(252, 267)	3829	(3764, 3890)	3763	(3703, 3822)
2024	282	(274, 290)	3894	(3821, 3963)	3825	(3758, 3891)
2025	286	(278, 295)	3967	(3886, 4043)	3882	(3808, 3953)
2026	278	(269, 287)	4051	(3961, 4134)	3942	(3862, 4021)
2027	305	(296, 315)	4124	(4026, 4215)	4019	(3932, 4103)
2028	316	(307, 327)	4194	(4089, 4293)	4081	(3988, 4172)
2029	312	(302, 323)	4291	(4177, 4397)	4146	(4046, 4243)
2030	307	(296, 318)	4368	(4246, 4481)	4220	(4114, 4324)

(b) Females

	Total aged 16-17		Total aged 18 and older		Total requiring care in an adult centre*	
Year	Number	95% prediction interval	Number	95% prediction interval	Number	95% prediction interval
2013	300	-	2440	-	2447	-
2014	258	-	2556	-	2533	-
2015	252	-	2651	-	2465	-
2016	253	-	2739	-	2743	-
2017	239	-	2798	-	2792	-
2018	256	(253, 260)	2869	(2848, 2889)	2883	(2864, 2902)
2019	241	(237, 246)	2928	(2897, 2959)	2955	(2928, 2982)
2020	233	(227, 238)	3012	(2971, 3051)	3029	(2994, 3063)
2021	258	(252, 265)	3051	(3002, 3099)	3116	(3075, 3157)
2022	240	(233, 248)	3121	(3063, 3178)	3170	(3123, 3218)
2023	235	(227, 243)	3182	(3115, 3246)	3245	(3192, 3299)
2024	262	(254, 271)	3229	(3155, 3302)	3321	(3262, 3381)
2025	272	(263, 281)	3280	(3197, 3360)	3383	(3318, 3449)
2026	262	(252, 271)	3351	(3260, 3439)	3448	(3377, 3519)
2027	296	(286, 306)	3407	(3309, 3503)	3535	(3459, 3613)
2028	315	(304, 326)	3463	(3357, 3567)	3608	(3526, 3691)
2029	299	(288, 310)	3549	(3435, 3661)	3679	(3591, 3769)
2030	272	(261, 283)	3619	(3497, 3739)	3761	(3667, 3856)

* Total number requiring care in an adult centre is the number aged 19 and older plus one third of the number aged 16, 17 or 18.
